# Computational Identification and Functional Predictions of Long Noncoding RNA in *Zea mays*

**DOI:** 10.1371/journal.pone.0043047

**Published:** 2012-08-16

**Authors:** Susan Boerner, Karen M. McGinnis

**Affiliations:** Department of Biological Science, Florida State University, Tallahassee, Florida, United States of America; Sun Yat-sen University, China

## Abstract

**Background:**

Computational analysis of cDNA sequences from multiple organisms suggests that a large portion of transcribed DNA does not code for a functional protein. In mammals, noncoding transcription is abundant, and often results in functional RNA molecules that do not appear to encode proteins. Many long noncoding RNAs (lncRNAs) appear to have epigenetic regulatory function in humans, including HOTAIR and XIST. While epigenetic gene regulation is clearly an essential mechanism in plants, relatively little is known about the presence or function of lncRNAs in plants.

**Methodology/Principal Findings:**

To explore the connection between lncRNA and epigenetic regulation of gene expression in plants, a computational pipeline using the programming language Python has been developed and applied to maize full length cDNA sequences to identify, classify, and localize potential lncRNAs. The pipeline was used in parallel with an SVM tool for identifying ncRNAs to identify the maximal number of ncRNAs in the dataset. Although the available library of sequences was small and potentially biased toward protein coding transcripts, 15% of the sequences were predicted to be noncoding. Approximately 60% of these sequences appear to act as precursors for small RNA molecules and may function to regulate gene expression via a small RNA dependent mechanism. ncRNAs were predicted to originate from both genic and intergenic loci. Of the lncRNAs that originated from genic loci, ∼20% were antisense to the host gene loci.

**Conclusions/Significance:**

Consistent with similar studies in other organisms, noncoding transcription appears to be widespread in the maize genome. Computational predictions indicate that maize lncRNAs may function to regulate expression of other genes through multiple RNA mediated mechanisms.

## Introduction

### Expanding Roles for RNA in Eukaryotic Genomes

A narrow interpretation of the central dogma would dictate that the ultimate contribution of RNA molecules to cellular function and phenotype is dependent upon their translation into proteins. Over recent decades, the central dogma has been extended to encompass emerging roles of RNAs that are functional as RNA molecules and are not known to be translated. Collectively, RNA molecules that are not predicted to encode for a functional protein are referred to as noncoding. Noncoding RNAs can include small RNAs, generally under 200 base pairs in length, and longer molecules, sometimes referred to as long noncoding RNAs (lncRNAs). Some lncRNAs are likely precursor molecules that are processed into small RNAs, while others function as intact, long molecules that participate in a range of regulatory roles.

### Long Noncoding RNAs in Mammalian Transcriptomes

Recent studies in mouse and human indicate that a substantial portion of transcribed sequences may be non-protein coding [1]–[4]. Notably, non-protein coding transcripts may represent a higher percentage of predicted transcribed sequences than protein coding transcripts in mammals. These findings have triggered discussion about the role of non-coding RNAs (ncRNAs), and whether they are functional molecules or indications of transcriptional noise. The comparison of 3,122 mouse long noncoding RNAs with orthologous sequences in human and rat revealed selective forces acting on this set of noncoding transcripts similar to selective forces acting on coding transcripts. This result led to the conclusion that the noncoding transcripts investigated were likely functional [5]. However, while some noncoding sequences have distinctive conservation, many characterized long noncoding RNAs lack conservation, but have apparent functions in the genome [6], [7]. Further analysis revealed that although small RNAs (miRNA and snoRNAs) are conserved, long noncoding RNAs may not be under the same selective pressure [7].

Long intergenic noncoding RNA (lincRNA) loci share similar chromatin states with protein coding transcribed loci, and a comparison of the codon substitution frequency of protein coding regions to intergenic transcribed regions and intergenic untranscribed regions showed similar levels of conservation for protein coding and intergenic transcribed regions [8]. Together, these results point to directed transcription of lincRNAs, as opposed to indiscriminate transcriptional events. Further support for the functionality of long noncoding RNAs could come from analysis of protein interactions with the RNA molecule. As several known lncRNAs have an epigenetic role through their interaction with chromatin remodeling complexes [9]–[13], an investigation into the interaction of lincRNAs with chromatin remodeling complexes was carried out. Approximately 3,000 lincRNAs were identified in human cells using the methods already developed in the mouse [8]. These lincRNAs were then analyzed for interactions with chromatin remodeling complexes. It was found that approximately 20 percent of the lincRNAs identified interacted with the polycomb repressive complex 2 (PRC2), and a smaller percentage with other chromatin remodeling complexes [14]. This predicts a substantial percentage of noncoding RNA with the potential to regulate chromatin structure.

### Long Noncoding RNA and the Regulation of Gene Expression

lncRNAs have the potential to regulate gene expression. This regulation is frequently sequence homology dependent, and the particular mechanism of regulation can be associated with homology to different regions of the regulated gene. Recently, a number of regulatory promoter associated lncRNAs have been characterized whose transcription block the transcription of proximal protein coding loci [15], [16]. Conversely, transcription of some promoter lncRNAs induce an open chromatin formation that facilitates activator binding and transcription of the associated protein coding gene [17].

Intronic lncRNA transcripts have also been implicated in a variety of regulatory roles, including the regulation of splicing events [18], and potential activity via small RNA regulatory pathways [19], [20]. An abundance of lncRNAs associated with the 3′ UTR of protein coding genes was noted in the analysis of full-length cDNAs in mouse [1], and an in depth study of 3′ UTR lncRNAs suggests a range of possible functional roles for these transcripts [21]. A specific example in Drosophila is the 3′ UTR of an RNA transcript of the Oskar gene that is required for oogenesis completion [22].

### Long Noncoding RNA in Plants

A comparatively small amount of lncRNAs have been identified in plants. One of the first was discovered in *Medicago truncatula*, Enod40. Further investigation revealed two roles for the RNA: as a peptide-encoding mRNA, and as a ncRNA molecule with a functional secondary structure [23], [24]. In addition, in *Arabidopsis thaliana*, evidence suggests that lncRNAs transcribed from the flowering locus C (FLC) are necessary for vernalization. One study found an antisense transcript, COOLAIR, to FLC that blocks transcription of the sense transcript [25]. Another study reported an intronic lncRNA, COLDAIR, originating within FLC that recruits PRC2 to epigenetically silence the locus [26].

While relatively few lncRNAs have been identified and characterized in plants, plant genomes encode the homologs for many chromatin remodeling proteins, including those known to interact with some lncRNAs in mammals (www.chromdb.org). Additionally, a distinctive pathway in plants utilizing noncoding RNA in epigenetic regulation was recently discovered. This pathway is RNA-directed DNA methylation (RdDM). Two RNA polymerases unique to plants, RNA polymerase IV (pol IV) and RNA polymerase V (pol V), are involved in the production of siRNAs which target loci for cytosine methylation and transcriptional regulation of effected loci. A substantive model for this pathway has been proposed, based upon genetic and biochemical data from model plant organisms like Arabidopsis and maize (reviewed in [27]). The model predicts at least two different types of lncRNAs, RNA pol IV and RNA pol V products, that may be detected in plants cells with an active RdDM pathway.

RdDM is important for maintaining silencing of transposons and repetitive elements [28], [29]. There is also evidence that this pathway is involved in flowering time in Arabidopsis through an antisense noncoding RNA transcribed by pol IV at the Flowering Locus C [30]. In maize, RNA-directed transcriptional silencing has been implicated in paramutation through studies of the mutant *mediator of paramutation 1* (*mop1*). Mop1 is orthologous to the Arabidopsis RNA dependent RNA polymerase that acts in RdDM, and is necessary for the establishment and maintenance of several examples of gene silencing [31]–[33].

Epigenomic analysis has been employed to characterize the maize genome, and identified three major categories of small RNAs, including predicted microRNAs (miRNAs), other predicted short hairpin forming RNAs (shRNAs), and predicted small interfering RNAs (siRNAs) [34]. According to current models, each of these small RNA categories require longer RNA precursors for their biogenesis. The shRNA category were classified based on the potential of their predicted precursors to form hairpin structures, and speculated to include currently unidentified miRNAs.The siRNAs would be predicted to generated by RNA-dependent RNA polymerase requiring pathways, including the RdDM pathway.

Collectively, these results suggest that noncoding RNAs may be an essential element of epigenetic gene regulation in plants, but relatively little is known about the abundance and activity of this type of molecule in plants.

### Computational Identification of Long Noncoding RNA and its Limitations

Several studies in a variety of species analyzed transcript sequence data with the intent to identify noncoding transcripts using computational approaches [35]–[39]. Typical steps in the computational identification of noncoding transcripts are to select for transcripts that either lack a complete ORF or have only a short ORF that lacks homology to known proteins. However, distinguishing coding from noncoding transcripts is complex [40], [41], due to transcripts that are short and protein coding, and functional long noncoding transcripts that contain long open reading frames with homology to known proteins [41]. Some noncoding transcripts in fact originate from protein coding loci [22], [24], [42], [43]. These challenges led to advances in computational methods [44], [45], and recent approaches have employed support vector machines (SVM) to distinguish between coding and noncoding transcripts [46], [47]. The use of machine learning algorithms, such as SVMs, has increased the accuracy of identification of protein coding potential to over 90 percent.

### A Computational Approach to Identifying Noncoding RNA in Plants

Plants are reliant on epigenetic gene regulation for proper growth and development, but little is known about the role of lncRNAs in such regulatory events in plants. Computational resources can be combined with existing genomic datasets to estimate noncoding RNA levels and predict functionality. This approach was applied to existing genomic data in the model crop plant *Zea mays*, resulting in the identification and classification of a collection of transcribed sequences in maize that do not appear to have protein coding potential, and may act as regulatory, noncoding RNAs.

## Results and Discussion

Two different methods were used to identify transcribed sequences that lack protein coding capacity. The first is a computational pipeline written specifically for this project in the programming language Python, and the second is a web-based, publicly available tool that utilizes an SVM [46].

### Developing and Optimizing a Python Pipeline

**Figure 1:**
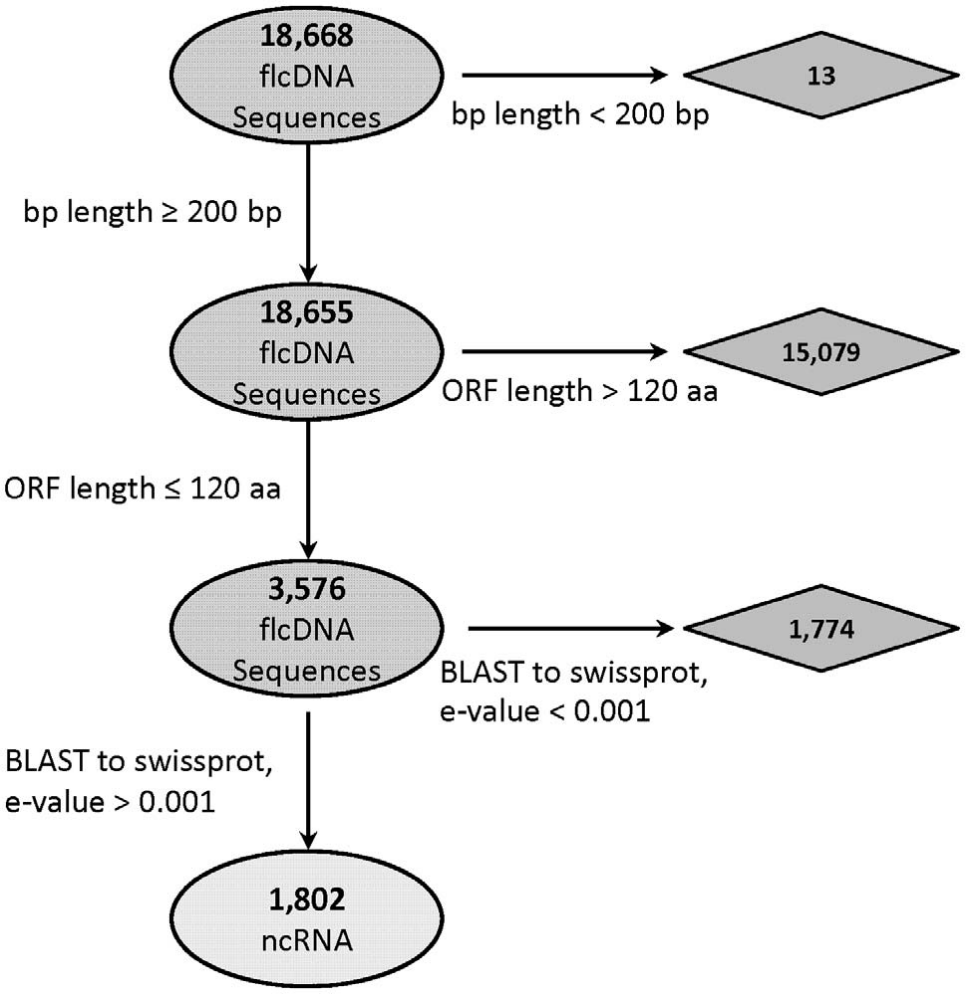
Python pipeline results on 18, 668 full-length cDNAs. The starting data set for the pipeline are 18,668 full-length cDNA sequences. The sequences are sequentially analyzed for protein coding potential using ORF length and homology of predicted protein with known proteins as indications of coding potential. The full-length cDNA sequences that pass through are designated as noncoding RNAs (ncRNAs) and are contained in Dataset S1 (supplementary data).

Three important criteria were used to discriminate noncoding transcripts based on previous studies [35]–[39]: transcript length, open reading frame (ORF) size, and homology with known proteins. The parameters assigned to these criteria were based upon empirical analysis of existing datasets and consideration of known properties of the maize genome. A computational pipeline was developed in the programming language Python and optimized to sort maize transcripts based upon these features (Figure 1).

#### Criterion 1: transcript length

For the characterization of long noncoding RNA, transcript length is a defining feature. Molecule length parameters are adjusted to include longer molecules that may be processed into siRNAs, but exclude the processed small RNAs themselves. The typical cutoff is 200 bp, based primarily on the established transcript lengths for classes of short RNAs such as micro (mi)RNAs [48], piwi interacting (pi)RNAs [49], promoter associated RNAs (PASRs) and terminator associated RNAs (TASRs) [36].

#### Criterion 2: Open reading frame length

It is expected that some noncoding RNAs may coincidentally contain partial or complete ORFs. It is impossible to completely discriminate a functional ORF from a non-functional one without assaying for the presence of the predicted protein, however, it is assumed that a large, complete ORF is likely to be translated into a protein. Therefore, the second step in the pipeline is to discard transcripts with an ORF greater than a designated threshold value. The initial value of 120 amino acids (aa) ORF selection size was based on the sizes of complete ORFs in the sense direction of 14 known lncRNAs (Table 1); only 3 have an ORF greater than 120 aa. Empirical testing was used to further refine this value. Simple statistical analysis of ORF sizes was performed on a well annotated group of maize protein coding transcripts downloaded from The Chromatin Database (www.ChromDB.org) [50]. This set of protein coding transcripts has been manually curated and is supported with experimental evidence, serving as a useful control in the analysis.

**Table 1:** Known lncRNAs with ORF size in amino acids.

lncRNA	ORF in aa
enod40	0
COLDAIR	34
COOLAIR_short	38
COOLAIR_long	43
linc1257	68
NRON	71
HOTTIP	86
NEAT1	97
RepA	105
HOTAIR	106
AIR	112
XIST	136
TSIX	151
KCNQ1QT	289

ORF size varied between 103 aa and 2,199 aa, with a mean of 381 aa in this protein coding data set (Figure S1). Fifteen protein coding transcripts fell below 120 aa in ORF size, all of which with an ORF size of 103 aa. To exclude these protein coding transcripts at this point in the pipeline, the amino acid size selection for the ORF would need to be 102. Considering the ORF results for known lncRNAs (Table 1), a size selection reduced to 102 aa would exclude 3 of the 14 transcripts.

To determine the effect of changing the ORF size parameter,11 known lncRNAs with ORFs smaller than 120 amino acids were analyzed by a script that selected for specific ORF sizes in a stepwise manner. When the size of ORF selection is decreased, the number of lncRNAs that pass through selection is reduced (Figure S2). Therefore, to maintain a significant level of stringency without losing an excessive number of possible lncRNA transcripts, the 120 aa cut off was selected, with the assumption that later steps in the pipeline would further refine the dataset by selecting against conserved protein coding transcripts with ORFs lower than 120 aa.

#### Criterion 3: Homology with known proteins

The presence of an ORF does not necessarily indicate that a transcript is translated, so homology with known proteins was used as another indication of protein coding potential. Transcripts were aligned to the Swissprot database [51], a manually annotated non-redundant database of experimentally supported protein sequences. The Swissprot database was used to avoid false positive scoring due to translations of unsupported pseudogenes present in other protein databases.

### Comparison of the Python Pipeline and Coding Potential Calculator on Test Data Sets

The Coding Potential Calculator (CPC) is a Support Vector Machine (SVM) that classifies transcripts into coding and noncoding groups based on 6 criteria of the input sequence [46]. These criteria are used to assess the quality, completeness, and homology of the ORF to proteins in UniProt Knowledgebase [51]. This tool was used in parallel with the described computational pipeline to identify the most inclusive set of noncoding RNAs.

To validate the Python pipeline and compare it with CPC, a set of 199 lncRNAs from www.lncrnadb.com and the set of 248 protein coding transcripts from ChromDB were run through each method of noncoding RNA detection. The Python pipeline identified 121 of 199 lncRNAs as noncoding, equivalent to an accuracy of 61 percent. The Coding Potential Calculator identified 189 of 199 lncRNAs as noncoding, equivalent to an accuracy of 95 percent (Figure S3). The 10 lncRNAs identified as coding by CPC were also identified as coding by the Python pipeline. This result suggests that each method is able to discriminate some, but not all, noncoding transcripts. For some experiments, more stringent identification may be desirable. In such cases, it would be appropriate to focus exclusively on those RNAs designated as noncoding by both approaches.

The protein coding transcript set was also analyzed with both computational tools. The Python pipeline identified all 248 transcripts as coding. The Coding Potential Calculator identified 198 of 248 as coding, equivalent to an accuracy of 80 percent (Figure S4).

These two tests convey the difficulty in distinguishing between coding and noncoding transcripts, which stems from the complexity of the transcripts themselves. For example, both methods were unable to identify p53 as noncoding. The lncRNA p53 is a long transcript (2,586 bp) with a long complete ORF (393 aa). It is therefore clear that a number of noncoding RNA transcripts will require laboratory bench experimental techniques, or a more sophisticated computational analysis, to verify their coding capacity.

### Identification of Maize ncRNAs

**Figure 2:**
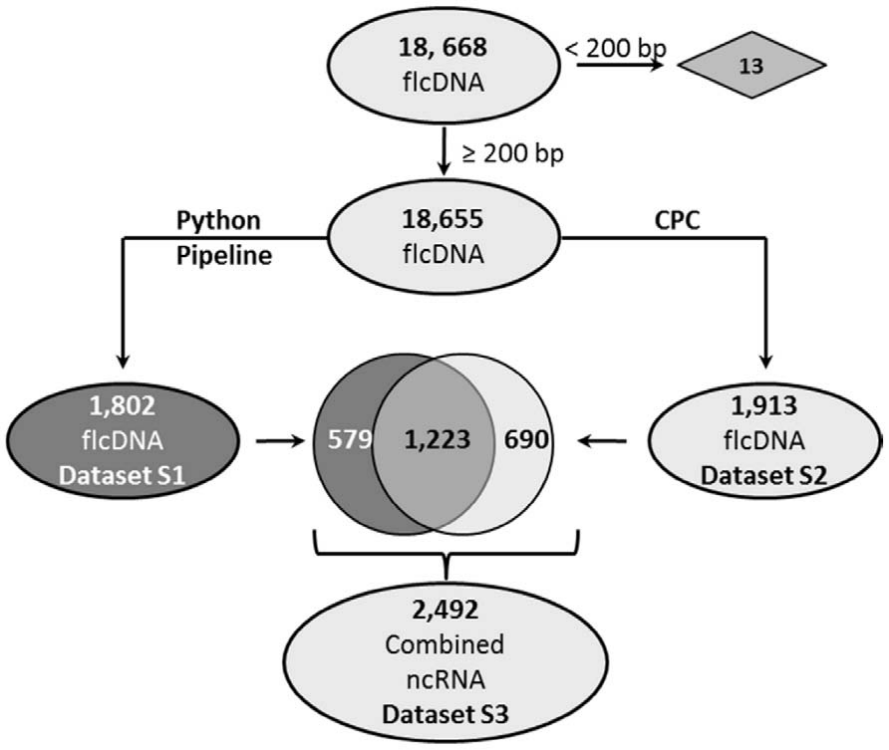
Combination of the Python Pipeline and CPC results. The Python Pipeline and CPC were used to identify noncoding RNAs, the number of transcripts identified as noncoding by each method is indicated. A subset were classified as noncoding by both methods, others were identified exclusively by one set or the other. The sequences identified as noncoding by either method were combined into one comprehensive set (Combined ncRNA). Datasets for ncRNAs identified by the Python pipeline, CPC, and the combined set were designated Dataset S1, S2, and S3 respectively (supplementary data).

To identify potential noncoding RNAs in maize, a set of sequences from the maize full length cDNA sequencing project [52] was analyzed. The starting dataset included 18,668 transcripts, and was analyzed in parallel with the Python pipeline and CPC. Of the original 18,668 full-length cDNA from maize, 1,802 were designated as noncoding by the Python pipeline (Figure 1). The largest number of transcripts were excluded at the ORF size step, reinforcing the importance of this parameter. Using CPC, 1,913 full length cDNAs from maize were identified as noncoding (Figure 2).

### Combination of the Python Pipeline and CPC Datasets

Among the potential lncRNAs identified by the Python pipeline and CPC, 1,223 were in common. These 1,223 potential lncRNAs may represent the most stringently selected dataset, and may be the most appropriate for intensive laboratory follow up. For our bioinformatic approach, we wanted to create a comprehensive set of potential lncRNAs for further analysis, so the two sets were combined, resulting in 2,492 lncRNA candidates (Figure 2).

The 2,492 lncRNA candidates represent 13.3 percent of the total number of starting sequences. This value is relatively low compared to the number of noncoding transcripts identified in the analysis of 25,159 FANTOM 2 full-length cDNAs in mouse [1], at 48.7 percent. This relatively low number could reflect different biological requirements for lncRNAs in maize compared to mammals, or it could be a reflection of limitations in the starting dataset. For example, the mouse full-length cDNA sequences were based on analysis of 246 different libraries comprised of over 30 different tissues at different time points in development. Relatively few full length cDNA libraries have been sequenced from maize, and this analysis included two different libraries representing 27 different tissue types and stress treatments. This would exclude noncoding transcripts expressed in other tissue types or developmental stages. The analysis in mouse was also able to capture very long transcripts (over 4 kb) and rare transcripts, which would facilitate the identification of some lncRNAs. Another factor is the construction of full-length cDNA libraries being dependent on the presence of a poly-A tail at the 3′ end of the transcripts [52], which is likely to enrich for RNA polymerase II (pol II) transcripts. In *Arabidopsis thaliana*, two additional polymerases, RNA polymerase IV and V, transcribe noncoding sequences involved in gene silencing via siRNA production, and pol V transcripts are apparently not polyadenylated (reviewed by [27], [53], [54]).

### Classification of Maize lncRNAs as sRNA Precursors

In plants, small RNAs 20–25 nucleotides in length are an important class of noncoding RNA for the regulation of gene expression, and can originate from longer transcripts that are processed by endonucleases like Dicer. These small RNAs can influence gene expression at both the transcriptional and post-transcriptional level, and are produced via distinct pathways in plants (reviewed by [55]). One anticipated fate of the lncRNA candidates would be to serve as precursor molecules that are processed into small RNAs. The 2,492 lncRNA candidates were characterized for small RNA precursor potential (Figure S5, Dataset S4, S5, S6, and S7), based upon homology with known small RNA sequences in maize.

Published small RNA sequences were compiled and used to populate databases for sequence alignments with the identified lncRNAs. One data set of smRNA sequences included the smRNA transcriptome [34], which includes predicted microRNAs (miRNAs), other predicted short hairpin forming RNAs (shRNAs), and predicted small interfering RNAs (siRNAs). Three separate databases were created for these categories to align with candidate lncRNAs. Maize miRNA sequences provided by miRBase [56], [57] and miRNAs from the small RNA transcriptome database [34] were combined into one miRNA dataset and queried with the ncRNA dataset.

**Figure 3:**
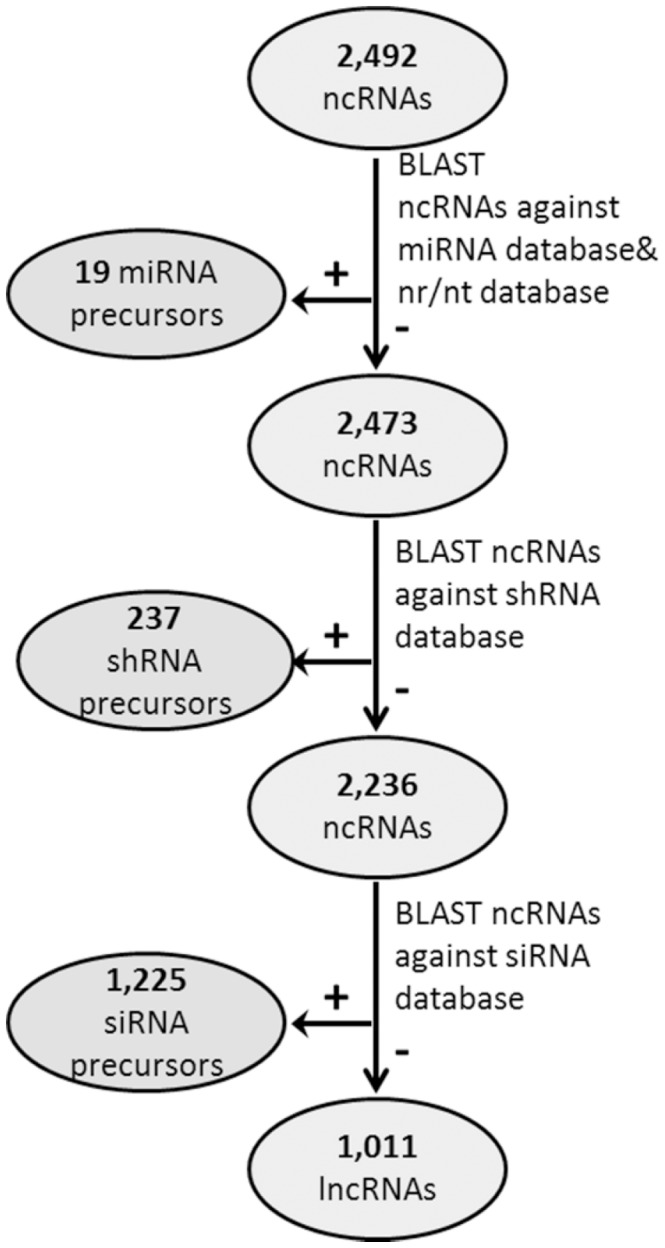
Results for the classification of combined noncoding RNA datasets (ncRNAs) based on small RNA precursor potential. Long noncoding RNA candidates identified above were classified into four different subsets based on alignment with small RNA databases including microRNA sequences (miRNA), short hairpin forming RNA sequences (shRNA),small interfering RNA sequences (siRNA), and those without homology to small RNA sequences, classified as long noncoding RNAs (lncRNA).

The 2,492 lncRNA candidates identified with CPC and the Python pipeline were sequentially aligned to the small RNA databases and classified according to the results (Figure 3 **)**. Twenty of the ncRNAs had homology with a miRNA sequence. Eighteen of these also aligned to an existing maize miRNA precursor in the non-redundant nucleotide database with varying levels of coverage and percent identity (Table 2), one had no significant homology with any other sequence, and one aligned to a maize protein coding mRNA. The ncRNA in this group that aligned to a maize protein coding gene was classified as a genic siRNA precursor, the remaining 19 were considered likely miRNA precursors.

**Table 2:** Homology of miRNA precursors with previously classified miRNA precursor sequences.

Full-length cDNA ID	Sequence with highest scoring alignment	% coverage	% identity	direction
Z27kG1_18025	gb|GQ905509.1| Zea mays clone zma-miR159a-2 precursor	99%	100%	sense
Z27kG1_17060	gb|GQ905546.1| Zea mays clone zma-miR168b precursor	90%	99%	sense
Z27kG1_17126	b|GQ905514.1| Zea mays clone zma-miR160a precursor	82%	100%	sense
Z27kG1_06696	gb|GQ905605.1| Zea mays clone zma-miR394b-2 precursor	71%	99%	sense
Z27kG1_12681	gb|GQ905528.1| Zea mays clone zma-miR166b-2 precursor	59%	100%	sense
Z27kG1_16032	gb|GQ905515.1| Zea mays clone zma-miR160b precursor	57%	100%	sense
Z27kG1_17306	gb|GQ905546.1| Zea mays clone zma-miR168b precursor	49%	99%	sense
Z27kG1_09597	b|GQ905553.1| Zea mays clone zma-miR169c precursor	47%	100%	sense
Z27kG1_08877	gb|GQ905563.1| Zea mays clone zma-miR169j-4 precursor	46%	100%	antisense
Z27kG1_22830	gb|GQ905563.1| Zea mays clone zma-miR169j-4 precursor	36%	100%	antisense
Z27kG1_23905	gb|GQ905575.1| Zea mays clone zma-miR171j_5prime-1 precursor	27%	100%	sense
Z27kG1_15711	gb|GQ905520.1| Zea mays clone zma-miR162-2 precursor	21%	100%	sense
Z27kG1_02106	gb|GQ905557.1| Zea mays clone zma-miR169h_3prime precursor	18%	100%	antisense
Z27kG1_02804	gb|GQ905543.1| Zea mays clone zma-miR167g_5prime precursor	17%	100%	sense
Z27kG1_08524	gb|GQ905590.1| Zea mays clone zma-miR319b_5prime-1 precursor	16%	100%	sense
Z27kG1_22328	gb|GQ905572.1| Zea mays clone zma-miR171f_3prime-1 precursor	12%	99%	sense
Z27kG1_19659	gb|GQ905533.1| Zea mays clone zma-miR166k/m precursor	10%	83%	sense
Z27kG1_02711	gb|GQ905546.1| Zea mays clone zma-miR168b precursor	7%	94%	sense
Z27kG1_09008	No hit to precursor	n/a	n/a	n/a
Z27kG1_07922	Zea mays indeterminate spikelet 1 (ids1) mRNA	92%	99%	sense

By alignment to the other small RNA databases, the remaining RNAs were classified as either small RNA precursors or lncRNAs. In total, 237 ncRNAs were classified as shRNA precursors, and 1,225 as siRNA precursors. The remaining 1,011 ncRNAs were classified as lncRNAs that are likely to function as longer molecules.

A total of 1,481 transcripts contained a small RNA sequence, which is equivalent to 59.4 percent of the total number of ncRNAs identified. This may reflect that small RNAs are important and abundant regulatory molecules in plants. While it may also be indicative of biased datasets, with over-representation of these types of ncRNAs, only miRNAs are known to be predominantly dependent upon pol II transcription in plants. Thus, we anticipate that any bias in the current dataset would underrepresent small RNA precursors due to the exclusion of non-polyadenylated molecules.

Due to the understood mechanisms of biogenesis, siRNAs are expected to derive from a longer molecule cleaved into multiple small RNAs by specific endonuclease activity. To determine if multiple small RNAs derived from miRNA, shRNA, and siRNA precursor ncRNAs, small RNAs were mapped to individual ncRNA molecules. Many small RNA precursor ncRNAs had homology to multiple small RNAs (Table 3). In many cases, ncRNAs had homology with multiple types of small RNAs (data not shown), suggestive of overlap between small RNA biogenesis pathways at many loci. In plants, there is evidence for overlapping epigenetic regulatory pathways (reviewed by [58], [59]). These results may indicate that some ncRNAs are processed into multiple types of small RNAs in maize and potentially act in multiple regulatory pathways.

**Table 3:** ncRNAs with homology to multiple small RNAs.

ncRNA type	No. transcripts	No. transcripts with homology to more than one small RNA
miRNA precursor	19	14
shRNA precursor	237	207
siRNA precursor	1225	724

### Genome Wide Localization of Long NoncodingRNAs

The region of homology between a regulatory RNA and its target gene is frequently predictive of the associated regulatory mechanism. ncRNAs with homology to the promoter region are frequently associated with transcriptional silencing mechanisms, while homology in coding regions may be indicative of post-transcriptional events, including mRNA degradation. The lncRNAs that are not precursors for small RNAs may function intact in regulatory capacities. Several of the characterized lncRNAs are transcribed from within a protein coding locus, and regulate the host gene via a variety of mechanisms. These regulatory lncRNAs thus share homology with their target genes in exonic, promoter, intronic, or other untranslated regions of genes.

The maize lncRNAs were localized within the genome relative to predicted protein coding genes (Figure S6, Dataset S8, S9, S10, S11, S12, S13, S14, and S15). First, the transcripts were sorted into two groups. The first group consisted of transcripts located within a gene model (genic) based upon alignment within a gene model from the maize protein evidence-based computationally predicted genes taken from the Filtered Gene Set (FGS) [60], the second group consisted of those transcripts located outside of these gene models (intergenic). For this analysis, a gene model consists of the untranslated regions (UTRs), exons, introns, and 500 bp up and downstream of these features.

**Figure 4:**
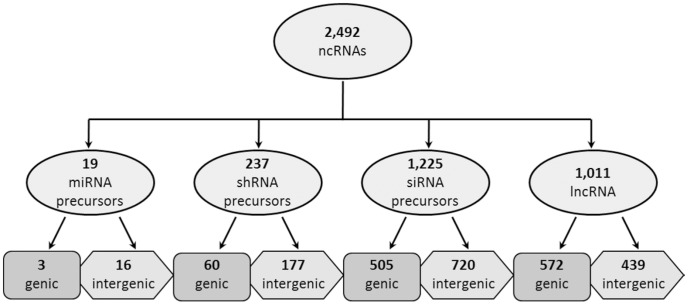
Results of genic vs. intergenic localization. Each subgroup of ncRNA was localized to either a genic or intergenic region in the maize genome. The number of genic and intergenic transcripts for each class of ncRNA are indicated.

Approximately 62% of the 1,481 small RNA precursor molecules were intergenic (Figure 4). This was consistent with the anticipated origin of these ncRNAs, because miRNA precursors tend to exist as a distinct gene in the genome that target protein coding genes after processing [55], while other categories of small RNAs are frequently associated with silencing of repetitive elements in intergenic regions [28], [29]. miRNA and shRNA precursors were particularly enriched for intergenic localization, with 85% and 75% of these sequences, respectively, mapping to intergenic regions. The few genic miRNA and shRNA precursors could represent intronic transcripts with regulatory roles, which have been detected in other studies [20]. The genic siRNA precursors represented a slightly higher percentage of the total siRNA precursors (41%), and this type of regulatory molecule might share sequence homology with one of several genic features depending on the type of regulation it is associated with [19], [61], [62].

The lncRNAs that do not appear to be small RNA precursors had homology with genic and intergenic loci in the genome, although slightly more (57%) appeared to be genic in origin. These genic lncRNAs could represent noncoding RNAs that negatively affect the transcription of their associated gene through transcriptional interference, as exemplified by COOLAIR in *Arabidopsis thaliana* [25]. Another possibility would be a noncoding transcript that recruits a chromatin modifying complex, such as PRC2, to their respective gene locus to establish silencing, like the intronic noncoding RNA COLDAIR, also identified in Arabidopsis [26]. This type of regulation is not restricted to an intronic region, however, and could be the result of a lncRNA associated with its gene host in any location, with varying mechanisms [63]. The intergenic loci might act as *trans*-acting RNAs to mediate expression of other loci, or could represent scaffolding transcripts associated with RdDM and heterochromatin formation in intergenic regions.

Genic transcripts were further localized within genes to gain insight into possible regulatory roles they may have on the associated FGS gene. The locations of the ncRNAs were based on the highest alignment score within the gene, and sorted further into the following categories: spliced over a gene, spans a gene from both the up and downstream region, spans a gene from both the 5′ and 3′ UTR, within 500 bp up or downstream of a gene, within a UTR, within an intron, and within the coding sequence of a gene (Figure S7). In addition, a distinction between the directionality of the ncRNA relative to its respective gene model, sense or antisense, was made.

To understand the relationship between ncRNAs and the associated filtered gene set gene models, the ncRNAs were separated into three groups based upon their subgenic localization and percent of gene model covered. The first group contained 541 noncoding transcripts that covered more than 98 percent of a gene model and those that are spliced over a gene; the former may span from the upstream to the downstream or from the 5′ to 3′ UTRs, the latter was not localized further. The second group contained 118 noncoding transcripts that covered a gene model between 50 and 98 percent; with this group it was determined whether the lncRNA candidates spanned to include a particular region. The third group included 481 ncRNAs with less than 50 percent coverage over a gene model; with this group a distinction was made between the lncRNA candidates located completely within a specific feature from those that spanned more than one feature.

**Figure 5:**
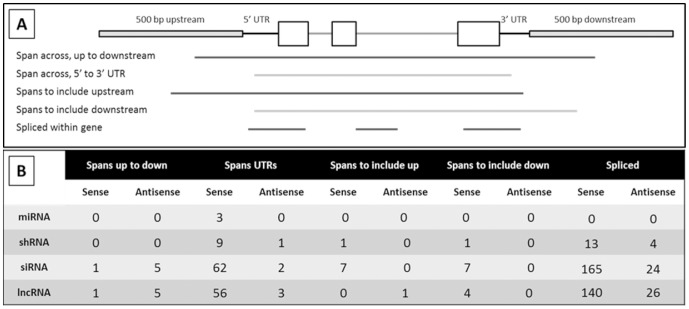
Results of genic localization for ncRNAs covering >98% of a gene model and spliced. A total of 541 ncRNAs were either spliced or had homology with filtered gene set genes over 98% of the gene model. This homology spanned different genic features (A). The number of sequences that aligned with a specific genic feature is indicated for each group of ncRNAs (B).

This analysis detected 470 sense transcripts with over 98 percent coverage of a predicted protein coding gene in the filtered gene set (Figure 5). Ten of these were randomly selected and manually analyzed. All ten of these gene models consisted of a single exon and had ORFs less than 120 aa with no significant homology to known proteins (data not shown). It is therefore likely that these transcripts have been mischaracterized as protein coding genes by genome annotation efforts. Spliced transcripts in the sense direction may also be associated with mischaracterized protein coding gene models. Both the high-coverage and spliced lncRNA candidates could therefore be intergenic functional lncRNAs. There is an established precedent for spliced, functional, lncRNAs. The long noncoding RNA database lists 40 of 178 lncRNAs as spliced transcripts, and two different search parameters to select for spliced and non-spliced transcripts on NRED (ncRNA Expression Database) revealed roughly 50 percent of the transcripts in the database as spliced [64], [65].

**Figure 6:**
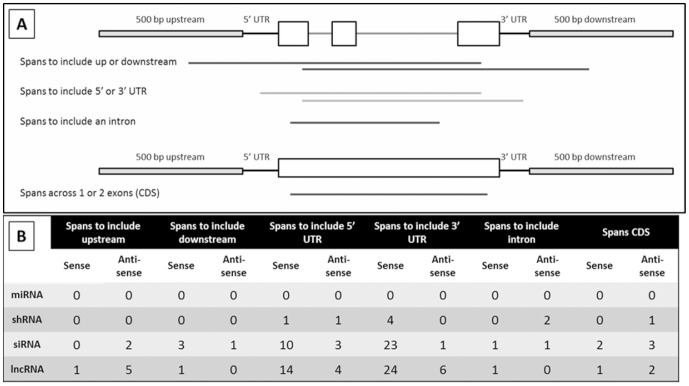
Results of genic localization for ncRNAs covering 50–98% of a gene model. A total of 118 ncRNAs were had homology with filtered gene set genes that covered 50–98% of the gene model. This homology spanned different genic features (A). The number of sequences that aligned with a specific genic feature is indicated for each groupof ncRNAs (B).

Of the 118 ncRNAs with 50–98% coverage of the associated gene model, 76 are sense transcripts which span the gene model to include either the 5′ or 3′ UTR (Figure 6). Several of these lncRNAs are associated with gene models that have long ORFs with significant homology to known proteins, yet themselves lack a complete ORF, leading to their classification as noncoding. These lncRNAs could be incomplete cDNAs that made it past the library screening and sequencing methods, or the result of transcription from an alternative start site within the gene model producing a lncRNA with a regulatory role within that gene locus. A study of noncoding RNAs that interact with the polycomb repressive complex 2 (PRC2) in human stem cells using a RIP-seq technique identified 4,446 RNAs that are sense, and 3,106 RNAs that are antisense, to annotated transcripts in the UCSC genome browser [66]. This result suggests that thousands of genic lncRNAs in the sense and antisense direction are functional and may act to recruit PRC2 to their gene locus for silencing in humans. PRC orthologs have been identified in Arabidopsis and maize [67], [68], and there is evidence that the polycomb group proteins contribute to gene regulation to control a variety of mechanisms in plants [69]. It is therefore possible that a subset of these lncRNA candidates function to regulate gene expression through their interaction with polycomb group proteins.

**Figure 7:**
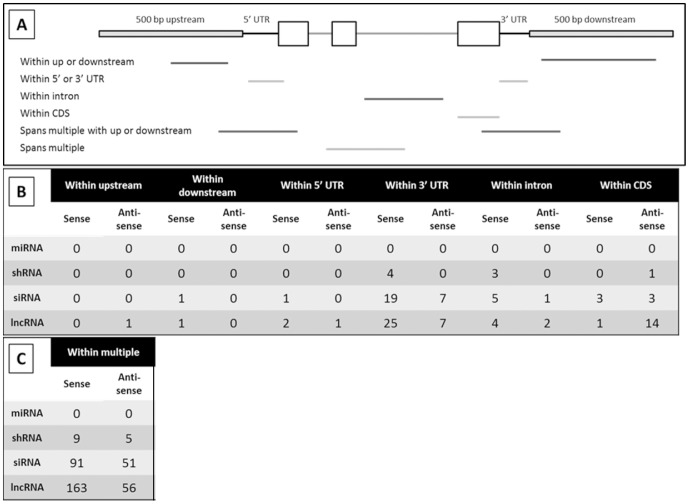
Results of genic localization for ncRNAs covering <50% of a gene model. A total of 472 ncRNAs were had homology with filtered gene set genes that covered less than 50% of the gene model. This homology spanned different genic features (A). (A). The number of sequences that aligned with a specific genic feature is indicated for each groupof ncRNAs (B). Some lncRNAs covered more than one feature of the gene (C). Within this group, those that include the upstream or downstream regions were reported separately.

There were 481 ncRNAs with less than 50 percent coverage over their associated gene model (Figure 7). Of these, a total of 236 mapped to the 3′ UTR alone or in combination with a 3′ UTR adjacent region in a protein coding gene model. There is evidence for functions of 3′ UTR associated lncRNAs in other species [21], [22], and many lncRNAs are associated with 3′UTRs in mice [1]. Gene regulation associated with 3′UTRs is not well understood, but this result suggests that there might be multiple examples of RNA-associated regulation of genes associated with 3′UTRs in maize.

There were 15 predicted lncRNA transcripts with <50% gene model coverage that mapped to the CDS of a gene model. Of these, 14 were antisense to the gene model. Antisense transcripts can mediate gene silencing via transcriptional or post-transcriptional mechanisms, with the latter involving mRNA degradation. Antisense -mediated mRNA degradation would likely generate siRNAs, and these were not detected in the available datasets. It is possible that these lncRNA candidates do in fact produce siRNAs that were not represented in the datasets used to create the databases for this study. Another function for these lncRNAs could be interference with or activation of pol II transcription along the gene in the sense direction, either through transcriptional interference, promoter mediated transcriptional silencing events or local changes in chromatin structure that inhibit or facilitate sense transcription.

### RdDM Related ncRNAs in Maize

One potential mechanism for small RNA biogenesis in plants is the RdDM pathway. Mop1 is a maize ortholog of Arabidopsis RDR2, an RNA-dependent RNA polymerase required for RdDM function. Mop1 is required for 24 nucleotide siRNA biogenesis in maize [70], and is thought to polymerize the complement of single stranded pol IV transcripts that are then processed into 24 nt siRNAs [27]. To determine if the ncRNAs detected by this study included any siRNA precursors associated with this gene silencing pathway, small RNA precursor ncRNAs were compared with a published dataset of Mop1-sensitive siRNAs [71]. A total of 282 ncRNAs were observed to include Mop1-sensitive siRNA sequences, of which 73 were genic and 209 intergenic in origin (Table 4). This is consistent with RdDM regulating the expression of repetitive intergenic elements and a subset of genes in the maize genome, which is in accord with genome wide expression profiles of maize Mop1 mutants [71]. Of the 73 genic ncRNAs with homology to Mop1-sensitive siRNAs, 46 were predicted siRNA precursors, 26 predicted shRNA precursors, and 1 a predicted miRNA precursor. Of the 209 intergenic ncRNAs with homology to Mop1-sensitive siRNAs, 110 were siRNA precursors, 97 shRNA precursors, and 2 miRNA precursors. Although some miRNAs appear to be dependent upon Mop1 activity [72] most are believed to be processed via mechanisms independent of RNA dependent RNA polymerase activity. The high percentage of mop1-sensitive small RNAs sharing homology with siRNA and shRNA precursors is therefore consistent with the current model of Mop1 activity in RdDM, and supports the idea that at least a portion of the shRNA precursors may not be unidentified miRNAs as originally predicted [34]. If the biogenesis of small RNAs from these molecules requires the activity of an RNA dependent RNA polymerase, any small hairpins formed by these molecules may not be required for processing into duplex small RNA molecules.

**Table 4:** ncRNAs with homology to Mop1-sensitive siRNAs.

ncRNA type	No. transcripts with homology to Mop1-sensitive siRNAs
Genic miRNA precursor	1
Genic shRNA precursor	26
Genic siRNA precursor	46
Intergenic miRNA precursor	2
Intergenic shRNA precursor	97
Integenic siRNA precursor	110

These results suggest that the ncRNAs detected by this study include ncRNAs generated by plant specific RNA polymerases involved in RdDM. Further analysis will be required to elucidate if these ncRNAs mediate transcriptional gene silencing events via a maize RdDM-like pathway.

### Sequence Composition of Maize lncRNAs

Another approach to gain insight into the possible functions of an RNA molecule is through analysis of its sequence characteristics. The length and base composition of an RNA molecule may provide insight about its overall structure, mechanism of biogenesis, or protein interactions. To further characterize lncRNAs in maize, sequences were analyzed to detect patterns in various sequence features. Transcript length and GC content were compared, no notable patterns exclusively associated with any category of RNA were detected by this analysis (data not shown).

### Repetitive Element Content of ncRNAs Based on Class and Location

Each group of lncRNA candidates based on class and location were run through Repeat Masker (www.repeatmasker.org) for repetitive element content analysis (Tables 5 and 6). The ChromDB protein coding set was analyzed again as a control for comparison; only 1.26 percent of this transcript set was masked as repetitive elements.

**Table 5:** Repetitive element content of genic lncRNA candidates by class.

	miRNA	shRNA	siRNA	lncRNA
Sequences	3	60	505	572
Total length (bp)*	4013	72968	511621	476532
GC level	49.84%	53.52%	53.14%	54.73%
Base pairs masked(% of total)	140 (3.49%)	9464 (12.97%)	28070 (5.49%)	12458 (2.61%)
	# elements (bases masked)	# elements (bases masked)	# elements (bases masked)	# elements (bases masked)
SINEs	0 (0)	0 (0)	3 (238)	3 (354)
LINEs	0 (0)	2 (659)	2 (599)	3 (1098)
LTRs	0 (0)	20 (5750)	52 (13320)	9 (1431)
DNA transposons	0 (0)	10 (1957)	34 (6013)	8 (1484)
Unclassified	0 (0)	1 (60)	8 (1100)	4 (563)
Small RNA	0 (0)	0 (0)	3 (238)	3 (354)
Satellites	0 (0)	0 (0)	0 (0)	0 (0)
Simple repeats	4 (115)	11 (418)	88 (4237)	81 (4100)
Low complexity	1 (25)	16 (620)	68 (2563)	112 (3248)
	Bases masked (% of total)	Bases masked (% of total)	Bases masked (% of total)	Bases masked (% of total)
Total interspersedrepeats	0 (0)	8426 (11.6)	21270 (4.2)	4930 (1)

* Total length, in base pairs, of all sequences analyzed.

**Table 6:** Repetitive element content of intergenic lncRNA candidates by class.

	miRNA	shRNA	siRNA	lncRNA
Sequences	16	177	720	439
Total length (bp)*	25993	258071	928982	434240
GC level	46.84%	51.6%	50.73%	51.39%
Bases masked(% of total)	2065 (7.94%)	88239 (34.19%)	117546 (12.65%)	16876 (3.89%)
	# elements (bases masked)	# elements (bases masked)	# elements (bases masked)	# elements (bases masked)
SINEs	0 (0)	1 (96)	2 (125)	2 (241)
LINEs	2 (204)	3 (344)	25 (9950)	9 (1377)
LTRs	4 (1325)	124 (70873)	174 (70266)	23 (5325)
DNA transposons	2 (350)	43 (10507)	115 (25314)	18 (3739)
Unclassified	0 (0)	12 (1016)	21 (5302)	9 (995)
Small RNA	0 (0)	5 (3431)	2 (125)	2 (241)
Satellites	0 (0)	0 (0)	10 (1453)	2 (156)
Simple repeats	5 (186)	23 (1088)	78 (4041)	57 (2410)
Low complexity	0 (0)	29 (980)	82 (3218)	78 (2633)
	Bases masked (% of total)	Bases masked (% of total)	Bases masked (% of total)	Bases masked (% of total)
Total interspersedrepeats	1879 (7.23%)	82836 (32.1%)	110957 (11.9%)	11677 (2.7%)

* Total length, in base pairs, of all sequences analyzed.

Within the genic set of ncRNAs, the shRNA precursors had the highest repetitive sequence content, with over 12 percent of the total sequences masked as repetitive elements (Table 5). The repetitive element content was generally low for miRNA precursors and lncRNAs that were localized to genic regions, at 3.49% and 2.62%, respectively (Table 5). This is also consistent with miRNAs originating from a unique miRNA precursor gene, and suggests that at least a subset of the shRNAs are not likely to represent currently unidentified miRNAs, although the shRNAs were originally classified on their potential to form miRNA-like hairpin structures [34].

The most abundant category of repetitive elements for genic and intergenic shRNA and siRNA precursors were annotated as long terminal repeat retrotransposons (LTRs) by RepeatMasker (Tables 5 and 6). LTRs have been demonstrated to be associated with pol V activity in Arabidopsis [54], which would be consistent with epigenetic regulation of these loci by RNA-mediated mechanisms. Long interspersed retrotranspons elements (LINEs) and DNA transposons were also abundant repetitive elements across this dataset. Generally, transposons are believed to be sources of ncRNAs that are important mediators of gene silencing (reviewed by [73]), and the DNA transposon-related sequences detected in this study may reflect this type of gene silencing mechanism in the maize genome.

The general trends were similar in the sequences that mapped to genic and intergenic regions (Tables 5 and 6), but the repetitive element content was higher for all categories of ncRNAs in the sequences that mapped to intergenic regions (Table 6). This is consistent with the structure of the maize genome, where repetitive elements are frequently associated with gene rich intervals but not typically within the gene models themselves [74]. Consistent with prior reports of a higher GC content within coding region DNA sequences [75], the intergenic lncRNAs also have a slightly lower GC content than the genic lncRNAs. This provides some additional evidence that these transcripts are indeed noncoding.

### Sequence Motifs of ncRNAs Based on Class and Location

Many regulatory events in the cell require the ability of nucleic acid polymers to bind to one another and with proteins in a sequence dependent manner. Transcription factors and chromatin modifying proteins are examples of this type of recognition. In many cases, a common sequence element can be associated with such interactions. In some cases, shared sequence motifs between different regulatory targets can also be detected, including those loci thought to be regulated by ncRNAs [76]. The maize ncRNAs detected in this study were analyzed to determine if there was evidence of sequence motifs that might be indicative of regulatory mechanisms.

Using Discriminative DNA Motif Discovery (DREME) [77], motifs were identified for the maize ncRNAs identified. DREME was developed specifically for the identification of transcription factor binding motifs from ChIP-seq data, and limits its search to motifs 8 base pairs in size to increase the speed of detection. No motifs were identified for miRNA precursors, possibly due to the small number of input sequences (2 genic, 17 intergenic), and only 6 were identified for the genic shRNA group (Figure S8). The ChromDB set of protein coding genes was analyzed as a control group to distinguish those motifs unique to the noncoding set. This analysis did not reveal specific motifs associated with ncRNA subgroups in this study, and most of the detected motifs were present in multiple classes of ncRNAs. In most groups of ncRNAs, motifs were identified that appeared similar to two motif types–a GAGA repeat like motif, and a CCACCA repeat like motif.

To distinguish motifs associated with repetitive elements, masked files were created for each class and location of lncRNA candidates and analyzed separately. Only 3 motifs were found in the genic shRNA candidate group with this analysis (Figure S8). The reduction of motifs in the masked sequences for shRNA candidates could be a result of the high level of repetitive element content in this class. The ten most significant motifs are reported for the other groups. When repetitive elements are masked, the CCACCA repeat like motif can be detected in all groups of ncRNAs. The functional significance of these motifs is not clear, and further analysis will be required to determine if these regions are involved in any protein-RNA or protein-DNA interactions.

## Materials and Methods

### The Starting Dataset

Full-length cDNA sequences were downloaded from www.maizecdna.org. The Unique Transcripts (UniTrans) from the 27 k strict PAVE assembly set were used, consisting of 24,467 transcripts of 2,294 contigs and 22,173 singletons [52]. Using a filter available on www.maizecdna.org, only transcripts that mapped to the genome at least once were downloaded, resulting in 18,669 sequences. Twenty potential contaminating sequences were identified during the analysis of the full-length cDNAs. These 20 sequences were also downloaded from www.maizecdna.org. Using a Python script, the 20 contaminating sequences were compared to the 18,669 sequences from the set that mapped to the genome. Only 1 contaminating sequence was present in the dataset and was removed. The remaining 18,668 sequences were used in this study.

### Designing the Python Pipeline

Open reading frame sizes for the 14 known lncRNAs were determined using Ugene (http://ugene.unipro.ru/) with the following parameters selected: Strand = Direct, Min length, bp = 363, Must terminate within region = true, must start with init codon = true. These settings selected for complete ORFs within each sequence in the forward strand. The longest ORF was reported.

The 248 well annotated protein coding genes in maize were taken from www.chromdb.org. A list of protein sequences for “confirmed” ChromDB genes was exported as a FASTA file using tools available on the website. The protein sequences in the FASTA file were then analyzed using Python scripts to determine ORF characteristics.

The validation of the ORF 120 amino acids (aa) cut by reducing the ORF cut size to 100 aa, 80 aa, and 40 aa, was performed using a combination of Ugene to determine ORF sizes and a Python script to select transcripts of the specified ORF size for the cut off.

### The Python Pipeline

A Python script was written that selected for transcripts greater than or equal to 200 bp in length, and generated a FASTA file of the results. These transcripts were then analyzed using Ugene for ORF status as described above. Another Python script was written to select transcripts with an ORF less than or equal to 120 aa and then compare these transcripts to protein sequences in the Swissprot database. The NcbiblastxCommandline wrapper from Bio.Blast.Applications was used in this script to BLASTX [78] each transcript against the Swissprot protein database with the following parameters: strand="plus”, db="swissprot”, max_target_seqs = 1, num_threads = 4, evalue = 0.001. The parameters for BLASTX were set to use only translations in the forward (“plus”) strand in the search. The transcripts with a translated region aligning with a protein sequence in the Swissprot database with an e-value above 0.001 were selected for and written to a new FASTA file.

### The Coding Potential Calculator

Full-length cDNA sequences greater than or equal to 200 bp (18,655) were uploaded to the CPC website (http://cpc.cbi.pku.edu.cn) for analysis. Parameters for the website were set to use only the forward strand. The output data was analyzed, and a list of the transcript IDs described as “noncoding” and “weakly noncoding” was created. A Python script was written to grab the transcript sequences associated with these IDs and write these transcripts to a new FASTA file.

### Small RNA Database Creation

Small RNA sequences were downloaded as FASTA files from NCBI under the series GSE15286 (http://www.ncbi.nlm.nih.gov/geo/query/acc.cgi?acc=GSE15286 ) [34]. The files for both root and shoot for each group (known_miRNA, shRNA, and siRNA) were combined into a single FASTA file. Using a Python script, the smRNA sequences in the FASTA file for each group were separated based on length into new FASTA files. For example, for the miRNA sequences, a separate FASTA file was created that contained all miRNA sequences 18 bp in length, another file for 19 bp miRNAs, another for 20 bp, 21 bp, 22 bp, and 23 bp. For the shRNA and siRNA sequences, the range in sizes was from 18 to 30 bp in length, a separate FASTA file created for each size. This separation based on size facilitated the mapping of the smRNAs to the lncRNA candidates using BLAST in a second Python script designed for this purpose (see below). NCBI-BLAST+ executables, version 2.2.23, was downloaded from the NCBI website for local use. Using the makeblastdb command available through the BLAST+ executables, BLAST nucleotide databases were created for the FASTA files described above.

### Python Script to Map smRNA Sequences to lncRNA

A Python script was created to BLAST the lncRNA candidates against each group of smRNAs (Program Script S1). The NcbiblastnCommandline wrapper from Bio.Blast.Applications was again used in this script. The parameters for BLASTN within the wrapper were set for a short DNA sequence match (dust="no”, perc_identity = 100, evalue = 1000, word-size = 7), with a script following to select for lncRNA candidates with an exact match to the complete smRNA sequence.

The miRNA precursors were analyzed further through a manual BLASTN of each sequence against the nucleotide collection (nr/nt) to look for similarity with characterized miRNA precursors in the database. The Program Selection was set to optimize for highly similar sequences (megablast); all other parameters were set at the default values.

### Genic vs. Intergenic Localization

Two BLAST databases were created for this analysis. The first consisted of genes from the Filtered Gene Set (FGS) of maize with protein evidence [60]. The Second database consisted of all chromosome assemblies for RefGen v2 of the B73 cultivar of maize. A FASTA file of gene models in the FGS that included 500 bp of sequence up and downstream of the gene (ZmB73_5b_FGS_genes_500.fasta.gz) was downloaded from maizesequence.org (http://ftp.maizesequence.org/current/filtered-set/). A Python script was written to pull out sequences from this file listed as having protein evidence in the info file available from the same website (ZmB73_5b_FGS_info.txt). This FASTA file was converted to a BLAST database using the mkblastdb command. To create the database of all the chromosome assemblies for maize, each chromosome assembly in FASTA format was downloaded from http://ftp.maizesequence.org/current/assembly/and combined into a single FASTA file. This FASTA file was converted to a BLAST database using the mkblastdb command. Both databases described above were used in the Python script written to localize lncRNA candidates in the genome (Program Script S2). Each group of lncRNA candidates (miRNA, shRNA, siRNA, and lncRNA) were analyzed separately using this script.

### Sub-genic Localization

For the sub-genic localization analysis, several BLAST databases were required containing specific regions of the FGS models. FASTA files for the databases were downloaded from maizesequence.org, except for the 5′ and 3′ UTR databases. For the intron database, the lower case lettering for introns within the pre-mRNA FASTA file (ZmB73_5b_FGS_pre_mrna.fasta.gz) was used to extract these sequences using a python script. A Python script was also written to extract the 500 bp regions up and downstream of gene models using the file including these regions (ZmB73_5b_FGS_genes_500.fasta). The FASTA files for the UTR databases were generated using BioMart available through GRAMENE (www.gramene.org/biomart). The BLAST databases created from these FASTA files were used in a Python script written to map lncRNA candidates within their associated FGS loci (Program Script S3). Each group of genic lncRNA candidates (miRNA, shRNA, siRNA, and lncRNA) were analyzed separately using this script.

### Sequence Analysis of lncRNA Candidates Based on Class and Location

A Python script was used to calculate the average length and the base pair content of lncRNA candidates for each class and location. The website www.repeatmasker.org was used to analyze the repetitive element content of the lncRNA candidates. The search engine used was abblast. The Speed/Sensitivity was set at default. The DNA source selected was Panicoid (maize, sugar cane, sorghum, millet). All other Advanced Options were set to the default parameters.

The DREME website was used to determine motifs in the lncRNA candidates (http://meme.sdsc.edu/meme/cgi-bin/dreme.cgi). The default parameters for all options were used in the search.

## Supporting Information

Figure S1 **Open reading frame sizes for 248 protein coding genes in Maize.** Open reading frame sizes are plotted for the set of 248 protein coding genes. The number of transcripts (y-axis) which had an ORF within each size range (x-axis) is depicted here.
(TIF)

Figure S2 **Validation of ORF size parameter.** Known lncRNAs with ORFs smaller than 120 amino acids were sequentially sorted by decreasing ORF size. The transcripts excluded by each amino acid cutoff (aa) is indicated.
(TIF)

Figure S3 **Known lncRNA test of the Python pipeline and CPC.** To test the accuracy of both methods in detecting coding vs. noncoding transcripts, a set of known lncRNAs were passed through each method individually (A). The results of both methods can be combined to identify the maximal number of noncoding RNAs (B).
(TIFF)

Figure S4 **Known protein coding test of the Python pipeline and CPC.** To test the accuracy of both methods in detecting coding vs. noncoding transcripts, a set of known protein coding mRNA transcripts were passed through each method individually (A). The results of both methods can be combined to identify the maximal number of protein coding RNAs (B).
(TIFF)

Figure S5 **Overview of the classification of combined noncoding RNA datasets (ncRNAs) based on small RNA precursor potential.** Strategy for identifying small RNA precursor potential in ncRNA dataset. A script was written and executed (Program Script S1), to parse transcript sequences from the ncRNA dataset into separate datasets designated as Dataset S4, S5, S6, and S7 (supplementary data), based upon small RNA precursor potential.
(TIF)

Figure S6 **Overview of the localization of ncRNAs.** ncRNA sequences were mapped to filtered gene set (FGS) to identify sequences that do (genic) or do not (intergenic) align with gene models in the FGS using a script developed for this analysis (Program Script S2). Genic and intergenic transcripts form each ncRNA category were grouped into separate datasets, designated as Dataset S8, S9, S10, S11, S12, S13, S14, and S15 (supplementary information).
(TIFF)

Figure S7 **Possible Locations of genic lncRNA candidates within a gene locus.** A script was developed and executed to determine the percent coverage and sub-genic location of ncRNA in protein coding loci (Program Script S3, supplemental information). Some genic transcripts covered a gene more than 98% or covered a smaller portion and were spliced (A). Other genic transcripts covered the gene model between 50 and 98% and could span one or more features within the gene, including untranslated regions (UTR), introns or exons (B). Still other genic ncRNAs covered less than 50% of a gene model; with this group a distinction was made between the lncRNA candidates located completely within a specific feature from those that spanned more than one feature and those that were contained within the coding sequence (CDS) (C).
(TIF)

Figure S8 **Sequence motifs identified for lncRNAs.** Most common sequence motifs found by DREME analysis of all ncRNAs (A). Most common sequence motifs found by DREME analysis of Repeat Masked ncRNAs (B). Motifs with similarity to two sequences that appeared to be shared between many detected ncRNAs are denoted with a * and ** respectively.
(TIFF)

Program Script S1 **Python script written to map small RNA sequences within lncRNA candidates using BLAST.**
(PY) Click here for additional data file.

Program Script S2 **Python script written to localize lncRNA candidates in the genome, either genic (within a gene model) or intergenic (outside of a gene model).**
(PY) Click here for additional data file.

Program Script S3 **Python script writtin to localize genic lncRNAs within a gene locus.**
(PY) Click here for additional data file.

Dataset S1 **The set of noncoding RNAs identified using the Python pipeline.**
(FASTA)

Dataset S2 **The set of noncoding RNAs identified using the Coding Potential Calculator.**
(FASTA)

Dataset S3 **The set of noncoding RNAs identified using both the Python pipeline and the Coding Potential Calculator.**
(FASTA)

Dataset S4 **The set of noncoding RNAs that contain a miRNA sequence.**
(FASTA)

Dataset S5 **The set of noncoding RNAs that contain an shRNA sequence.**
(FASTA)

Dataset S6 **The set of noncoding RNAs that contain an siRNA sequence.**
(FASTA)

Dataset S7 **The set of noncoding RNAs that do not contain a small RNA sequence from our database.**
(FASTA)

Dataset S8 **The set of noncoding RNAs that contain a miRNA sequence and map to a gene locus.**
(FASTA)

Dataset S9 **The set of noncoding RNAs that contain a miRNA sequence and map outside of a gene locus.**
(FASTA)

Dataset S10 **The set of noncoding RNAs that contain an shRNA sequence and map to a gene locus.**
(FASTA)

Dataset S11 **The set of noncoding RNAs that contain an shRNA sequence and map outside of a gene locus.**
(FASTA)

Dataset S12 **The set of noncoding RNAs that contain an siRNA sequence and map to a gene locus.**
(FASTA)

Dataset S13 **The set of noncoding RNAs that contain an siRNA sequence and map outside of a gene locus.**
(FASTA)

Dataset S14 **The set of noncoding RNAs that do not contain a small RNA sequence from our database and map to a gene locus.**
(FASTA)

Dataset S15 **The set of noncoding RNAs that do not contain a small RNA sequence from our database and map outside of a gene locus.**
(FASTA)
