# Supplementary figures and images for: Computational Identification and Functional Predictions of Long Noncoding RNA in Zea mays
Source: PLoS One. 2012 Aug 16;7(8):e43047. doi: 10.1371/journal.pone.0043047 (PMC3420876; doi:10.1371/journal.pone.0043047)

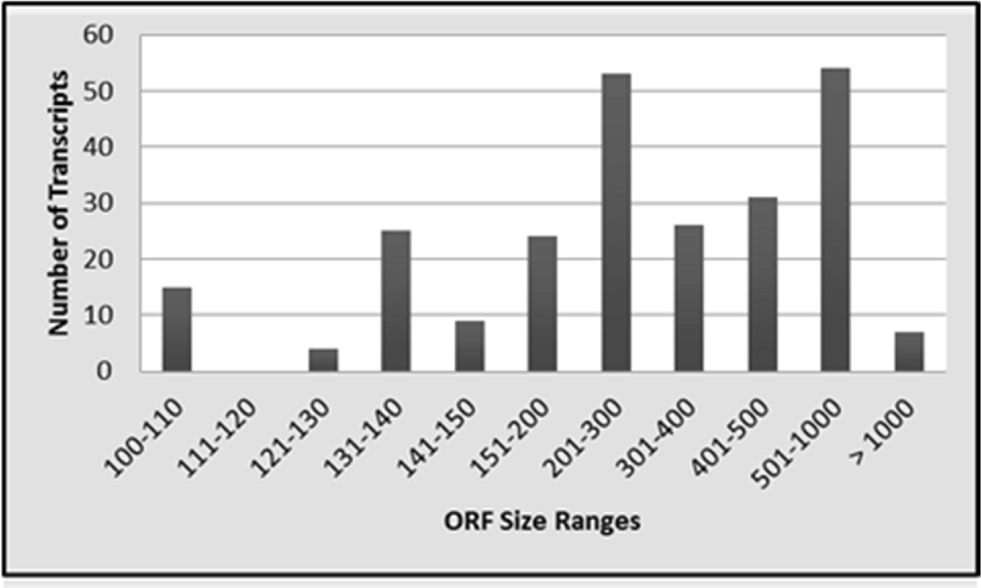

Supplement: Figure S1 — Open reading frame sizes for 248 protein coding genes in Maize. Open reading frame sizes are plotted for the set of 248 protein coding genes. The number of transcripts (y-axis) which had an ORF within each size range (x-axis) is depicted here. (TIF) [file pone.0043047.s001.tif]

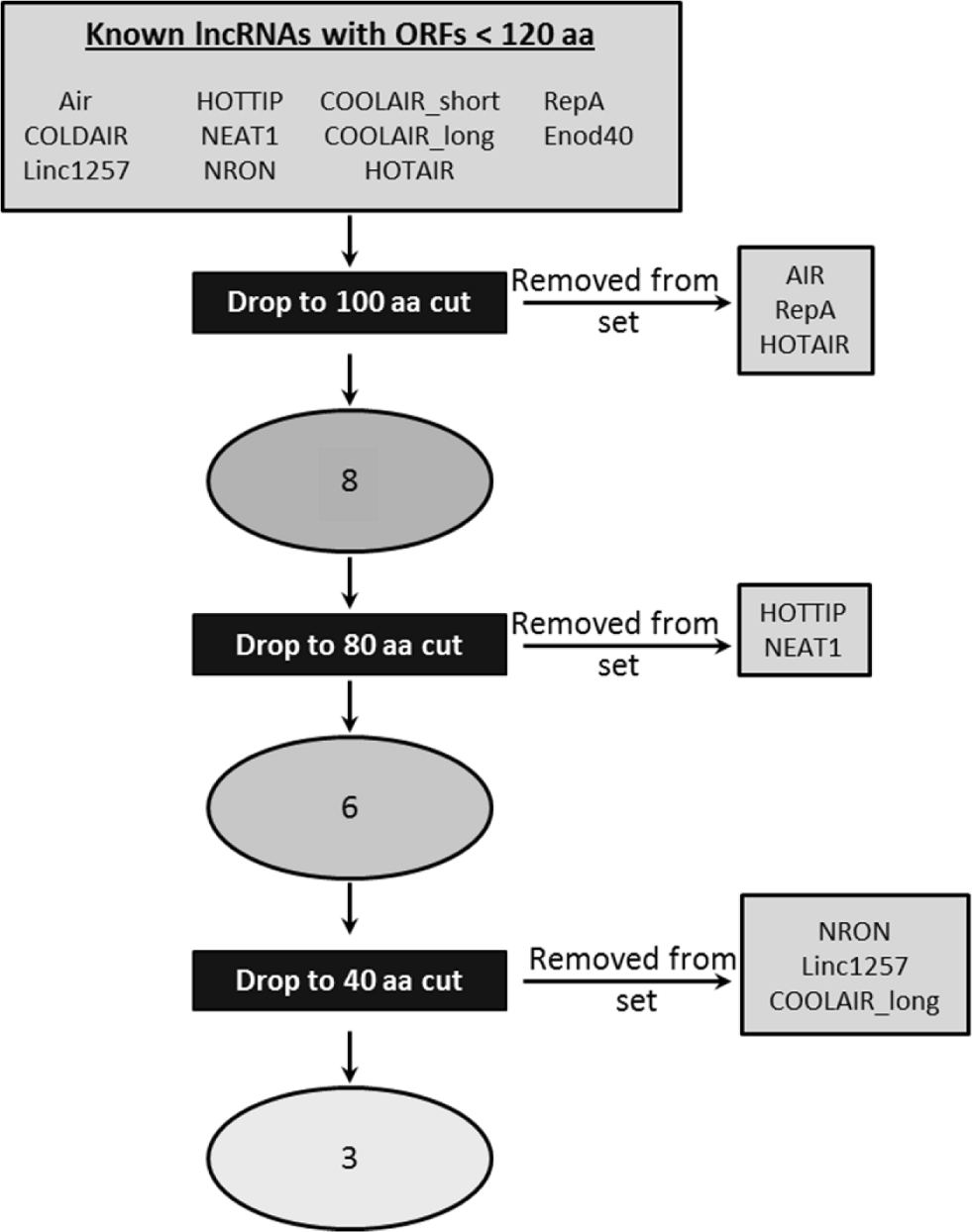

Supplement: Figure S2 — Validation of ORF size parameter. Known lncRNAs with ORFs smaller than 120 amino acids were sequentially sorted by decreasing ORF size. The transcripts excluded by each amino acid cutoff (aa) is indicated. (TIF) [file pone.0043047.s002.tif]

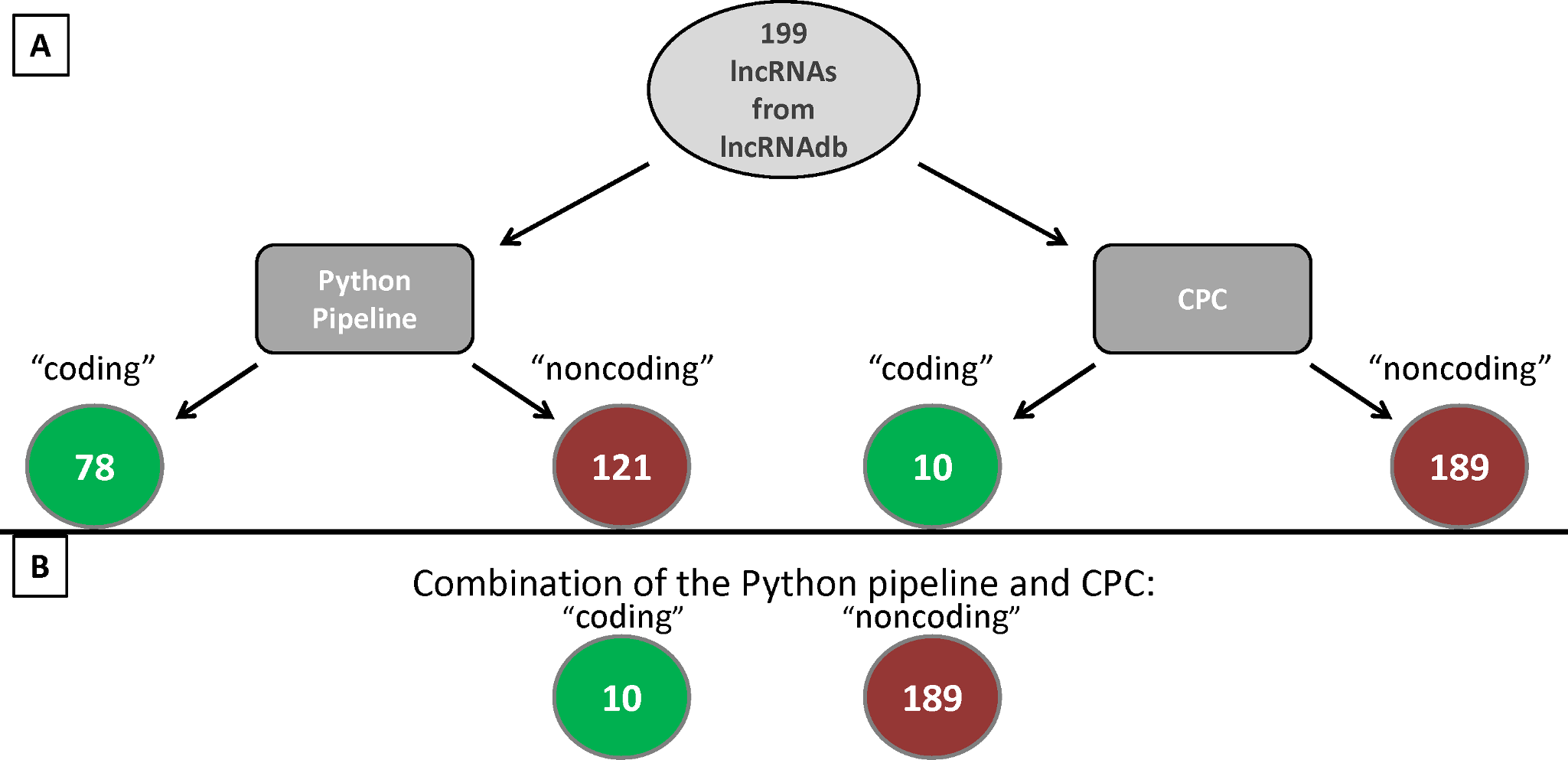

Supplement: Figure S3 — Known lncRNA test of the Python pipeline and CPC. To test the accuracy of both methods in detecting coding vs. noncoding transcripts, a set of known lncRNAs were passed through each method individually (A). The results of both methods can be combined to identify the maximal number of noncoding RNAs (B). (TIFF) [file pone.0043047.s003.tiff]

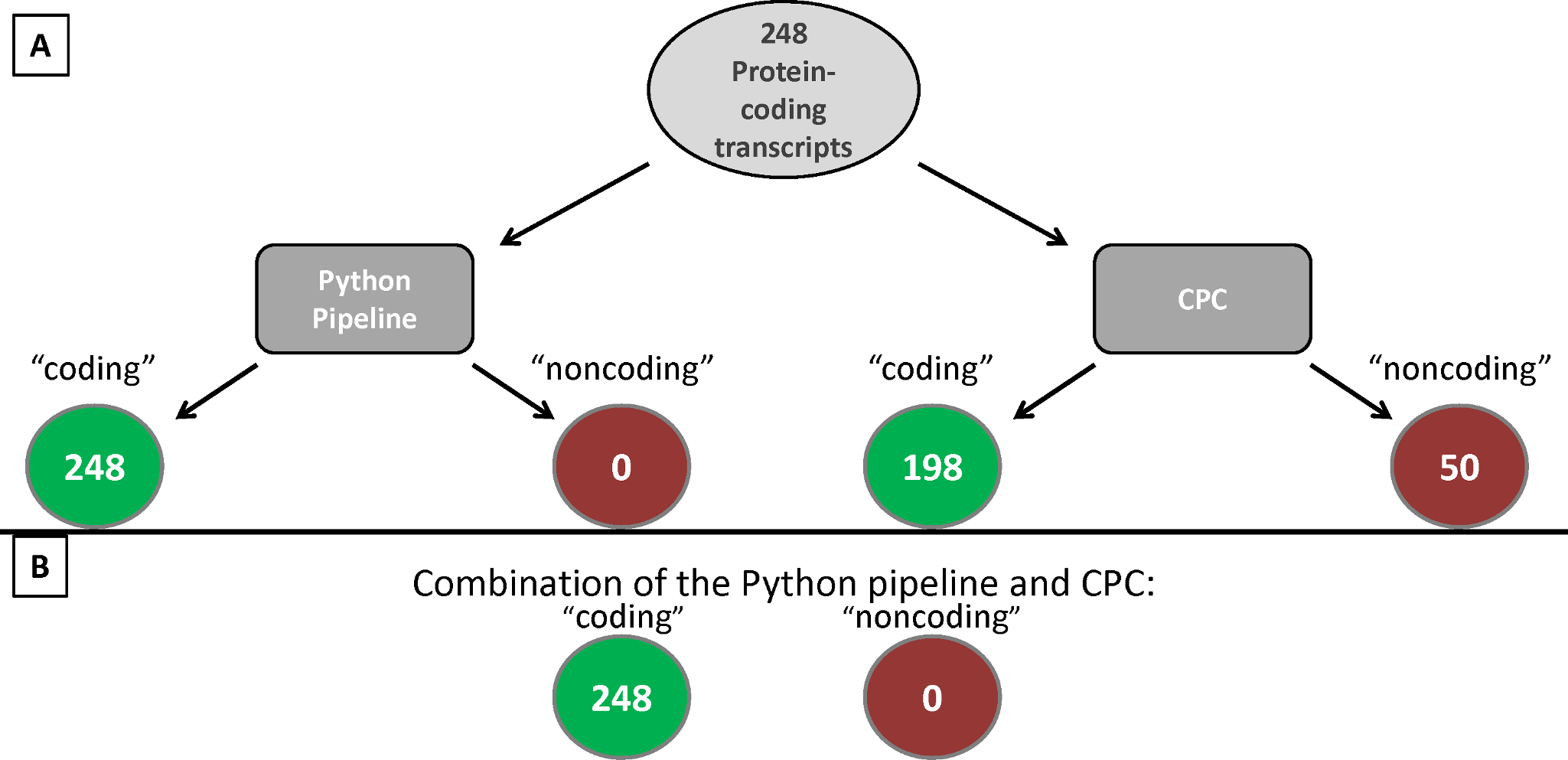

Supplement: Figure S4 — Known protein coding test of the Python pipeline and CPC. To test the accuracy of both methods in detecting coding vs. noncoding transcripts, a set of known protein coding mRNA transcripts were passed through each method individually (A). The results of both methods can be combined to identify the maximal number of protein coding RNAs (B). (TIFF) [file pone.0043047.s004.tiff]

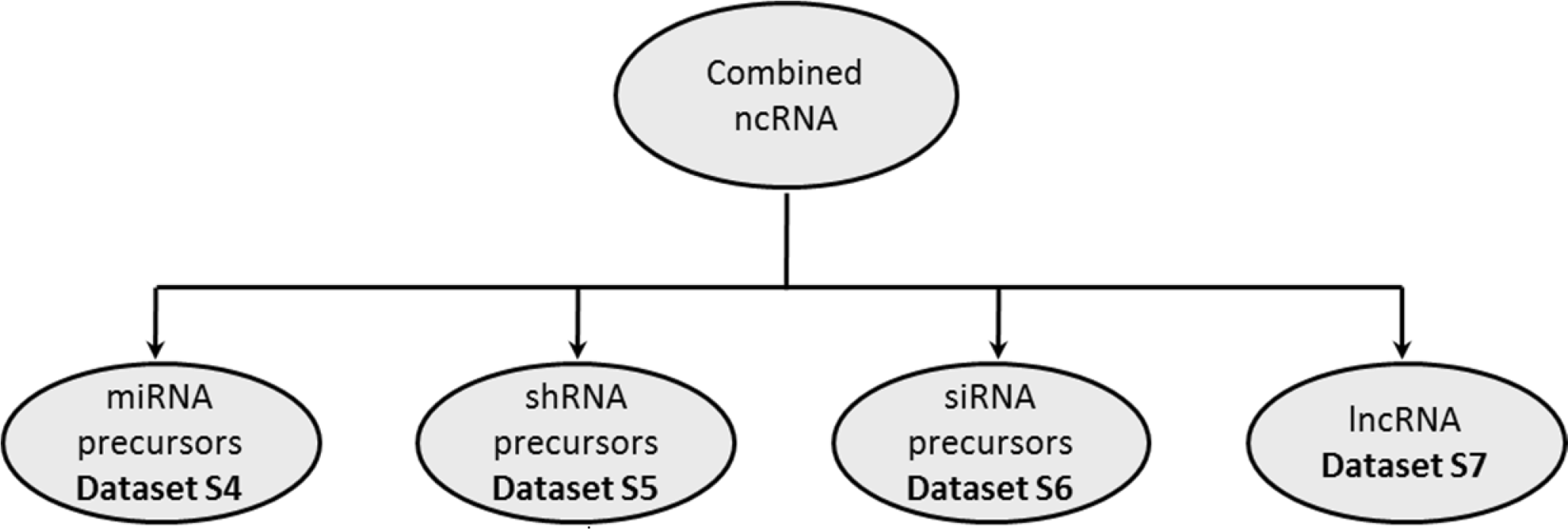

Supplement: Figure S5 — Overview of the classification of combined noncoding RNA datasets (ncRNAs) based on small RNA precursor potential. Strategy for identifying small RNA precursor potential in ncRNA dataset. A script was written and executed (Program Script S1), to parse transcript sequences from the ncRNA dataset into separate datasets designated as Dataset S4, S5, S6, and S7 (supplementary data), based upon small RNA precursor potential. (TIF) [file pone.0043047.s005.tif]

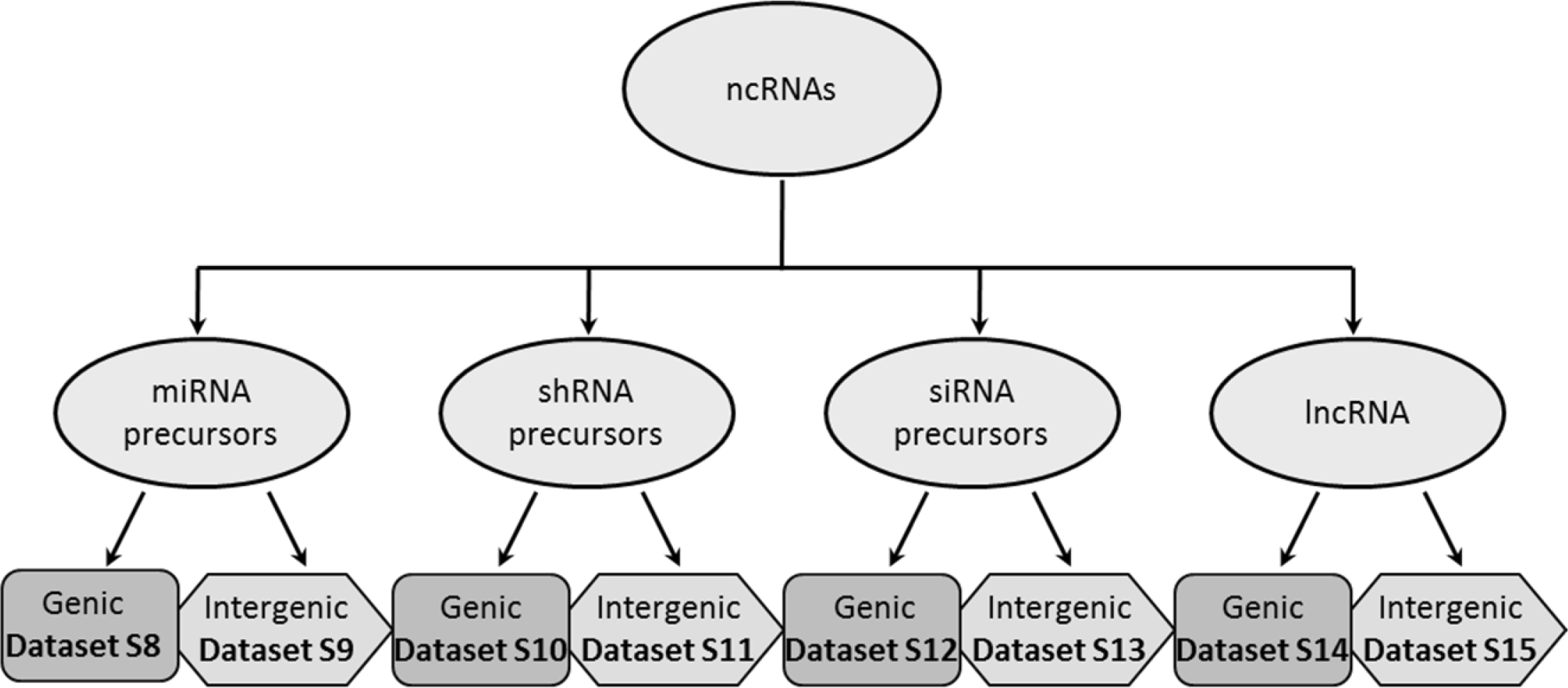

Supplement: Figure S6 — Overview of the localization of ncRNAs. ncRNA sequences were mapped to filtered gene set (FGS) to identify sequences that do (genic) or do not (intergenic) align with gene models in the FGS using a script developed for this analysis (Program Script S2). Genic and intergenic transcripts form each ncRNA category were grouped into separate datasets, designated as Dataset S8, S9, S10, S11, S12, S13, S14, and S15 (supplementary information). (TIFF) [file pone.0043047.s006.tiff]

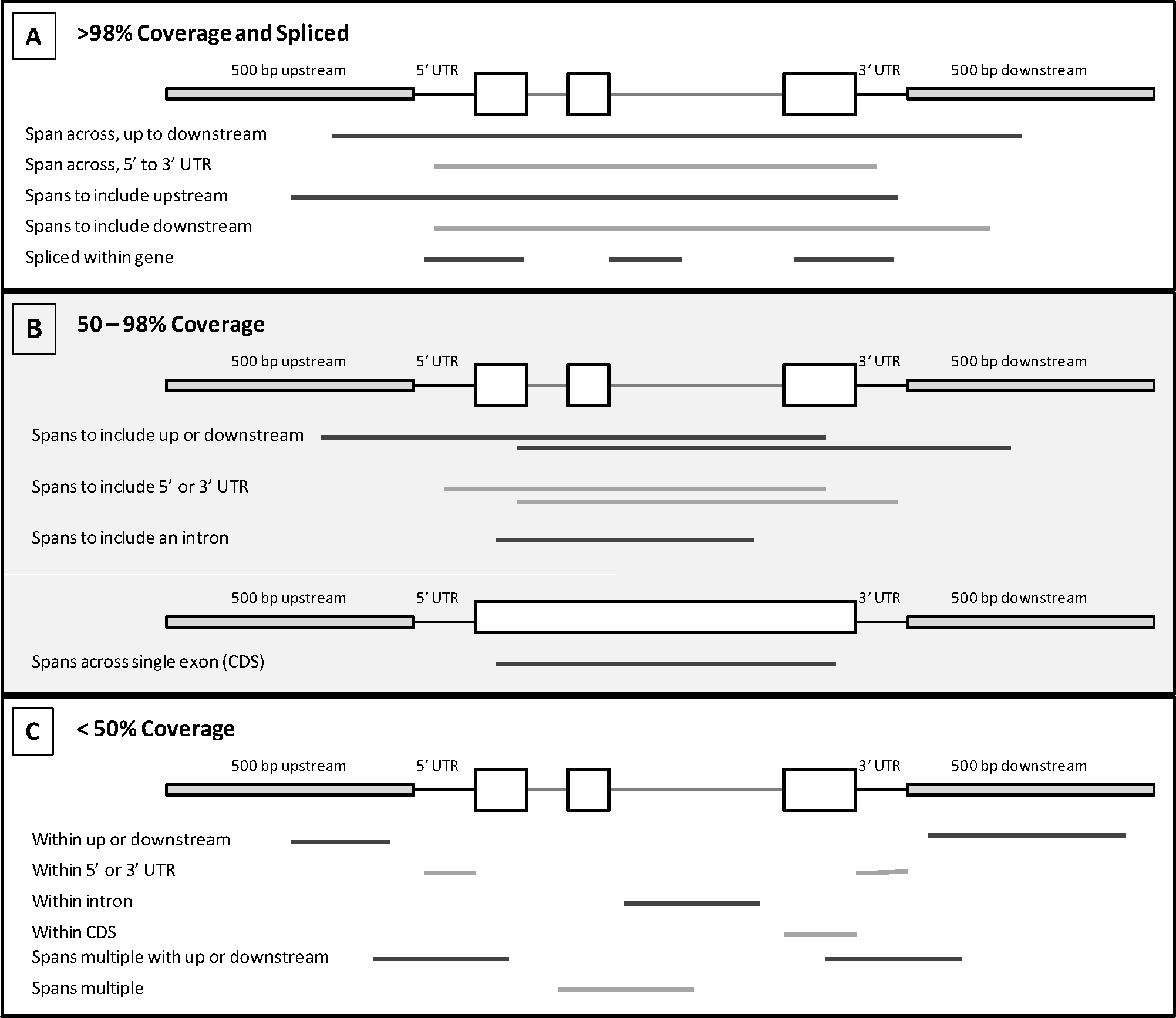

Supplement: Figure S7 — Possible Locations of genic lncRNA candidates within a gene locus. A script was developed and executed to determine the percent coverage and sub-genic location of ncRNA in protein coding loci (Program Script S3, supplemental information). Some genic transcripts covered a gene more than 98% or covered a smaller portion and were spliced (A). Other genic transcripts covered the gene model between 50 and 98% and could span one or more features within the gene, including untranslated regions (UTR), introns or exons (B). Still other genic ncRNAs covered less than 50% of a gene model; with this group a distinction was made between the lncRNA candidates located completely within a specific feature from those that spanned more than one feature and those that were contained within the coding sequence (CDS) (C). (TIF) [file pone.0043047.s007.tif]

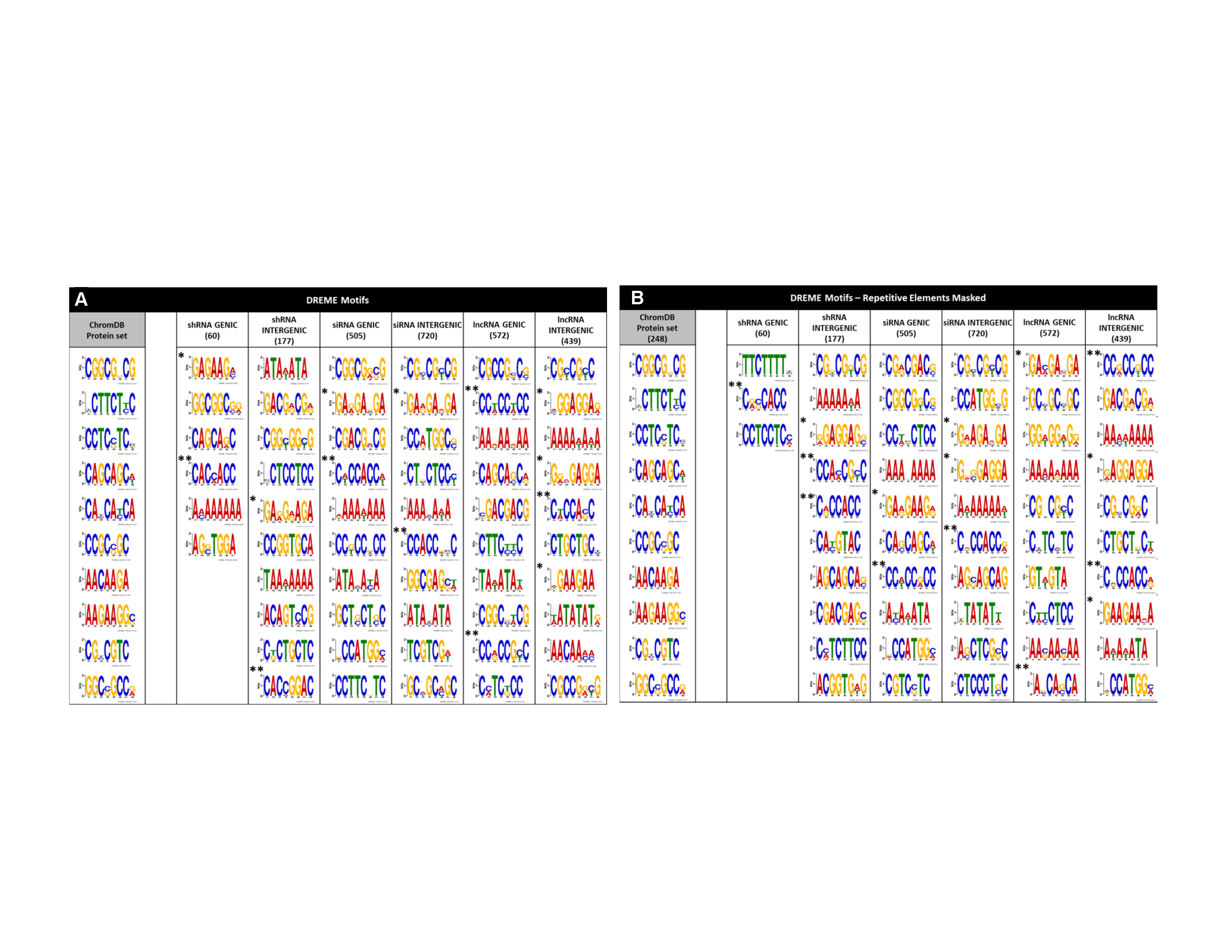

Supplement: Figure S8 — Sequence motifs identified for lncRNAs. Most common sequence motifs found by DREME analysis of all ncRNAs (A). Most common sequence motifs found by DREME analysis of Repeat Masked ncRNAs (B). Motifs with similarity to two sequences that appeared to be shared between many detected ncRNAs are denoted with a * and ** respectively. (TIFF) [file pone.0043047.s008.tiff]
